# Microstructural Model of Indacenodithiophene-*co*-benzothiadiazole Polymer: π-Crossing Interactions and Their Potential Impact on Charge Transport

**DOI:** 10.1021/acs.jpclett.3c02305

**Published:** 2023-09-27

**Authors:** Hesam Makki, Colm A. Burke, Alessandro Troisi

**Affiliations:** † Department of Chemistry and Materials Innovation Factory, University of Liverpool, Liverpool L69 7ZD, U.K.

## Abstract

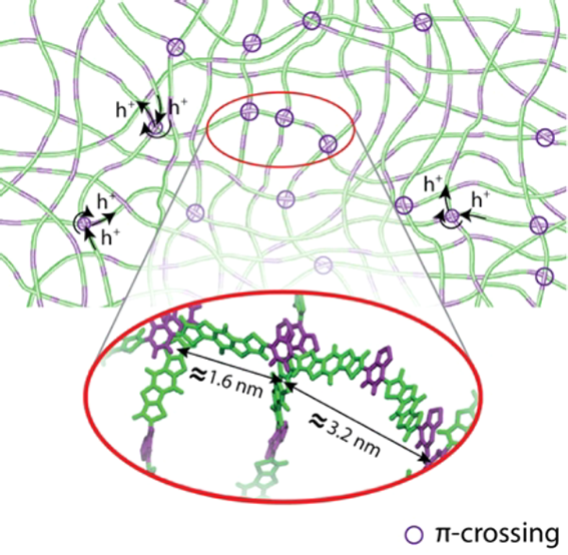

Morphological and electronic properties of indacenodithiophene-*co*-benzothiadiazole (IDTBT) copolymer with varying molecular weights are calculated through combined molecular dynamics (MD) and quantum chemical (QC) methods. Our study focuses on the polymer chain arrangements, interchain connectivity pathways, and interplay between morphological and electronic structure properties of IDTBT. Our models, which are verified against GIWAXS measurements, show a considerable number of BT-BT π–π interactions with a (preferential) perpendicular local orientation of polymer chains due to the steric hindrance of bulky side chains around IDT. Although our models predict a noncrystalline structure for IDTBT, the BT-BT (interchain) crossing points show a considerable degree of short-range order in spatial arrangement which most likely result in a mesh-like structure for the polymer and provide efficient pathways for interchain charge transport.

The emergence of semiconducting polymers (SCPs) with outstanding charge carrier mobility and without semicrystalline morphology has been a new paradigm in SCP development.^1−3^ IDTBT has been one of the most interesting,^3−5^ with respect to properties, e.g., great solubility and high charge carrier mobility,^4,6^ and puzzling, regarding the morphology–property relationships, SCPs of this kind. Therefore, the special structural features of this polymer have been extensively investigated.^7,8^ It is generally believed that the torsion-free backbone of polymer (due to the high torsion barrier between IDT and BT) results in a ribbon-like chain conformation and provides a great intrachain charge transport.^2,4,6,7,9−13^ However, there is a considerable degree of uncertainty in the reported characteristics of the IDTBT microstructure. For instance, while several studies emphasize its amorphous-like structure,^4,5,8,14^ some report a limited degree of crystallinity^2,6,9^ and there is also reported evidence of remarkable short- and medium-range order and unconventional packing (compared with conventional semicrystalline polymers).^13^ Thus, the degree and nature of order (if it exists) seem rather unclear. More importantly, there is no reported firm evidence about the origin of the observed (limited degree of) molecular arrangements in this polymer. Furthermore, the charge transport mechanism in IDTBT is (generally) believed to be mainly one-dimensional and through the backbone of polymer.^2,6,7^ This is mainly based on the lack or very limited amount of crystallinity as observed by DSC^14^ or GIWAXS^5,14^ for this polymer. However, it is believed that for the extraordinary high mobility of this polymer, a strong interchain electronic coupling is crucial.^15^ Nevertheless, due to uncertainties regarding the microstructure, the interchain charge transport pathways have not been elucidated.

MD simulations have traditionally been the method of choice for building models of the microstructure of polymers to be compared with the available experimental evidence.^16−23^ For IDTBT there is no consensus yet on the microscopic model with a paper predicting a planar conjugated backbone (IDT-BT torsional angles Φ_DT-BT_ of 5.2 ± 4.0°)^4^ and another suggesting a more twisted chain structure (Φ_IDT-BT_ = (25 ± 7)°–16°),^11^ a very critical distance as the planarity of the backbone was originally advanced as a key to explain the extraordinary properties of IDTBT in terms of a “ribbon-like” structure.

In this paper, we show the results of atomistic MD simulations and QC/MD calculations on model IDTBT to reconcile the various (and sometimes contrary) observations regarding the IDTBT microstructure with the focus on (i) the microstructure of polymer, existence, and origin of order (ii) interchain connectivity pathways, and (iii) the interplay between morphology and bulk electronic structure properties (as previous models focused on individual chain conformation and no in-depth interchain arrangement analysis has been performed previously^4,11,24^).

Figure 1a shows the IDTBT16 monomer (where 16 is the number of carbon atoms in each side chain) and polymer structures with 5, 10, and 20 repeating units. Note that the molecular weight of IDTBT16-20 (≈ 26 000 g/mol) is in the range of experimentally synthesized polymers.^7,25^ Accordingly, three simulation boxes consisting of 100 IDTBT16-5, 50 IDTBT16-10, and 25 IDTBT16-20 chains were constructed (see Supporting Information (SI) section 1 for force field parameters and model details). Annealing MD simulations (based on a sub-*T*_g_ equilibration protocol, suitable for MD polymer equilibration^26,27^) were employed to equilibrate polymer melts (see Figure 1b, and SI section 2 for details). The average end-to-end distance *L*_e_ of polymers in the simulation box was monitored during equilibration (Figure 2c). As shown, the required number of annealing cycles for equilibration of polymers considerably increases with chain length so that at least 60 annealing cycles (>1500 ns simulation time) are needed to obtain an equilibrated IDTBT16-20 model. It should be noted that the average *L*_e_ for equilibrated IDTBT16-20 is in quantitative agreement with the predicted *L*_e_ value (dashed line in Figure 1c) based on the worm-like chain model^28^ and the simulation of shorter oligomers, confirming the good equilibration of the model (see SI section 2 for details of this analysis). Also, as indicated in Figure 1c, IDTBT16-5 shows fully stretched chains in the equilibrated state (*L*_e_/*L*_c_ = 1.00, where *L*_c_ is polymer contour length), while longer polymer chains (i.e., 10- and 20mer) are not fully stretched.

**Figure 1:**
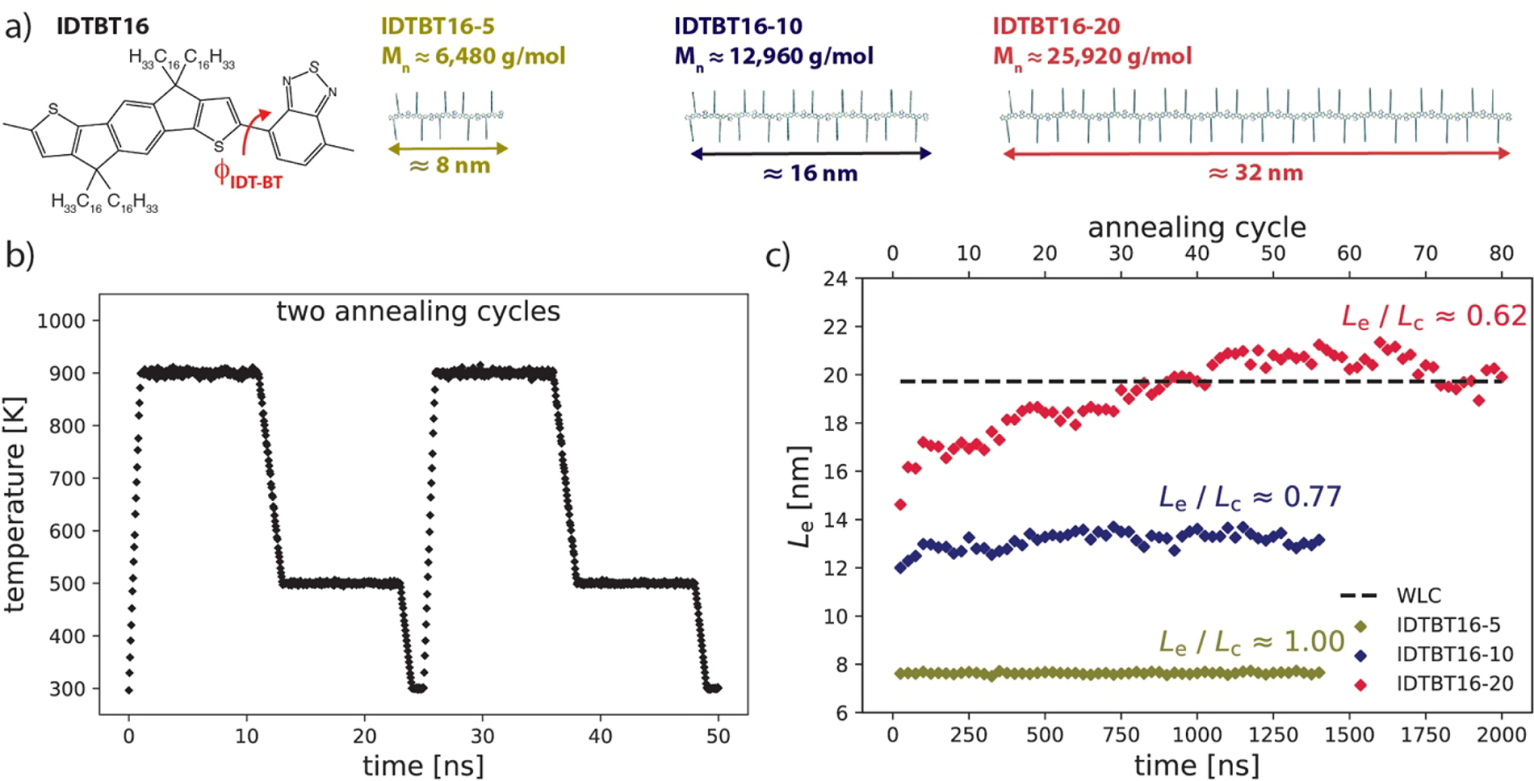
(a) IDTBT16 monomer and polymer models with different molecular weights (*M*_n_). (b) Two consecutive annealing cycles based on a sub-*T*_g_ equilibration protocol (the simulation *T*_g_ of IDTBT was estimated around 510 K, see SI section 2). (c) End-to-end distance (*L*_e_) of polymer as a function of time/annealing cycles and the normalized L_e_ by polymer contour lengths (L_c_). The dashed line shows the calculated L_e_ value for IDTBT16-20 based on the worm-like chain model (see SI section 2 for calculations).

**Figure 2:**
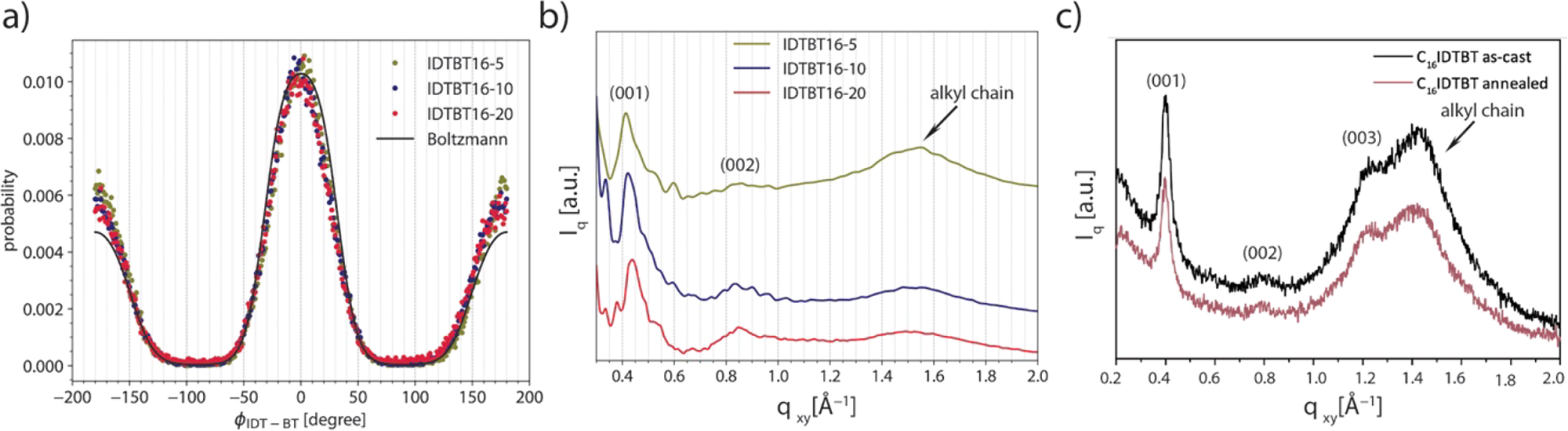
(a) IDT-BT torsional angle distribution for different models and the Boltzmann distribution based on the DFT-driven potential at 500 K. MD distributions are obtained from Φ_IDT-BT_ of all IDT-BT pairs in the polymer chains during 5 ns of the 500 K part of the last annealing cycle. (b) Simulated X-ray scattering patterns of three polymer models. (c) Experimental GIWAXS in-plane X-ray scattering pattern.

Figure 2a depicts the Φ_IDT-BT_ (torsional angle between IDT and BT, see Figure 1 a) distributions as obtained from MD simulations and the Boltzmann distribution based on the (DFT-derived) torsional potential (see SI section 1), both at 500 K. The Φ_IDT-BT_ distribution as obtained from our models shows two maxima at 0° and 180° and a rather wide torsional distribution (≈ ±30° at half-height of both peaks) suggesting a not significantly planar backbone. We also note a very good match between (the peak position and the width of peaks of) the Boltzmann distribution and those obtained from the MD models, indicating that the backbone conformation is little affected by specific intermolecular interaction, and therefore, it does not influence the intrachain transport.

We also used simulated X-ray scattering, as a powerful tool in calculating structural factors of polymer models,^29−31^ to further verify our bulk models (see SI section 3 for details). Figure 2b and 2c illustrate similar features in the scattering patterns as obtained from our models and GIWAXS^8^ experiment. In both cases, the presence of “backbone” reflections (00l), at ≈0.4 and 0.8 Å^–1^, which is a feature associated with the polymer repeat unit length (≈1.6 nm), and the broad peak around 1.5 Å^–1^ attributed to diffraction from disordered side chains^13^ can be seen.

Counting the number of π-stacking interactions per monomer (using the definition proposed in ref (27) and with criteria explained in SI section 2), we find that there is less than 0.005 IDT-IDT π–π interaction per IDT while about 10% of BT fragments are in π–π interactions in all models (see Figure 3a). The lack of π–π interactions for IDT is most likely due to the steric hindrance of bulky side chains around it. The simultaneous evidence of BT-BT and lack of IDT-IDT π–π interactions suggests that polymer chains are not aligned in parallel, as is usually the case for stacked chains in semicrystalline polymers. Thus, we calculated the relative angle between local director vectors θ_ij_ of chains which have BT pairs in π–π interactions (see Figure 3b where θ_ij_ is illustrated for two interacting chains). Figure 2b shows the total θ_ij_ distribution for all BT pairs in π–π interactions in IDTBT16-5, IDTBT16-10, and IDTBT16-20, during 100 ns of simulation (through 4 annealing cycles) in comparison with the distribution found for completely random vectors, proportional to sin(θ_ij_). A preferred orientation with θ_ij_ > 60° is clearly seen, suggesting that bulky side chains attached to IDT force chains to form BT-BT π–π interactions with a local relative perpendicular orientation. Note that these BT crossings have been previously observed for IDTBT and similar SCPs with low degrees of energetic disorder.^32^ Furthermore, TEM results^13^ show “ordered” regions overlap with a more-or-less uniform relative angle distribution above 20°—with two (slightly) preferential angles at about 20° and 90° (similarly, two small peaks at these angles can be seen in Figure 3b). Although θ_ij_ (as calculated here) is the relative angle between BT molecules and represents a very local orientation of chains as compared to TEM results, which give a relative angle between a few nanometer regions, the two observations consistently indicate that π–π interactions in IDTBT do not lead to parallel chain stacking as often observed for semicrystalline polymers. Also, we have confirmed that the side chain repulsion is the origin of this particular feature by constructing (hypothetical) IDTBT models with shortened side chains (see SI section 2). We observed that as the side chain lengths are shortened, the number of π–π interactions increases and the distribution of θ_ij_ tends toward 0°. This reconfirms that the reason behind the relative perpendicular orientation of BT-BT crossings is the steric hindrance of the bulky side chains. In addition, we observed that shortening the side chains (from 16-carbon to 1-carbon length) has a negligible effect on torsional angle distribution of the chains (see Figure S13 in SI). However, it results in a considerable degree of anisotropy (Figure S14) due to the packing of chains in crystalline domains, a common observation in semicrystalline polymers.

**Figure 3:**
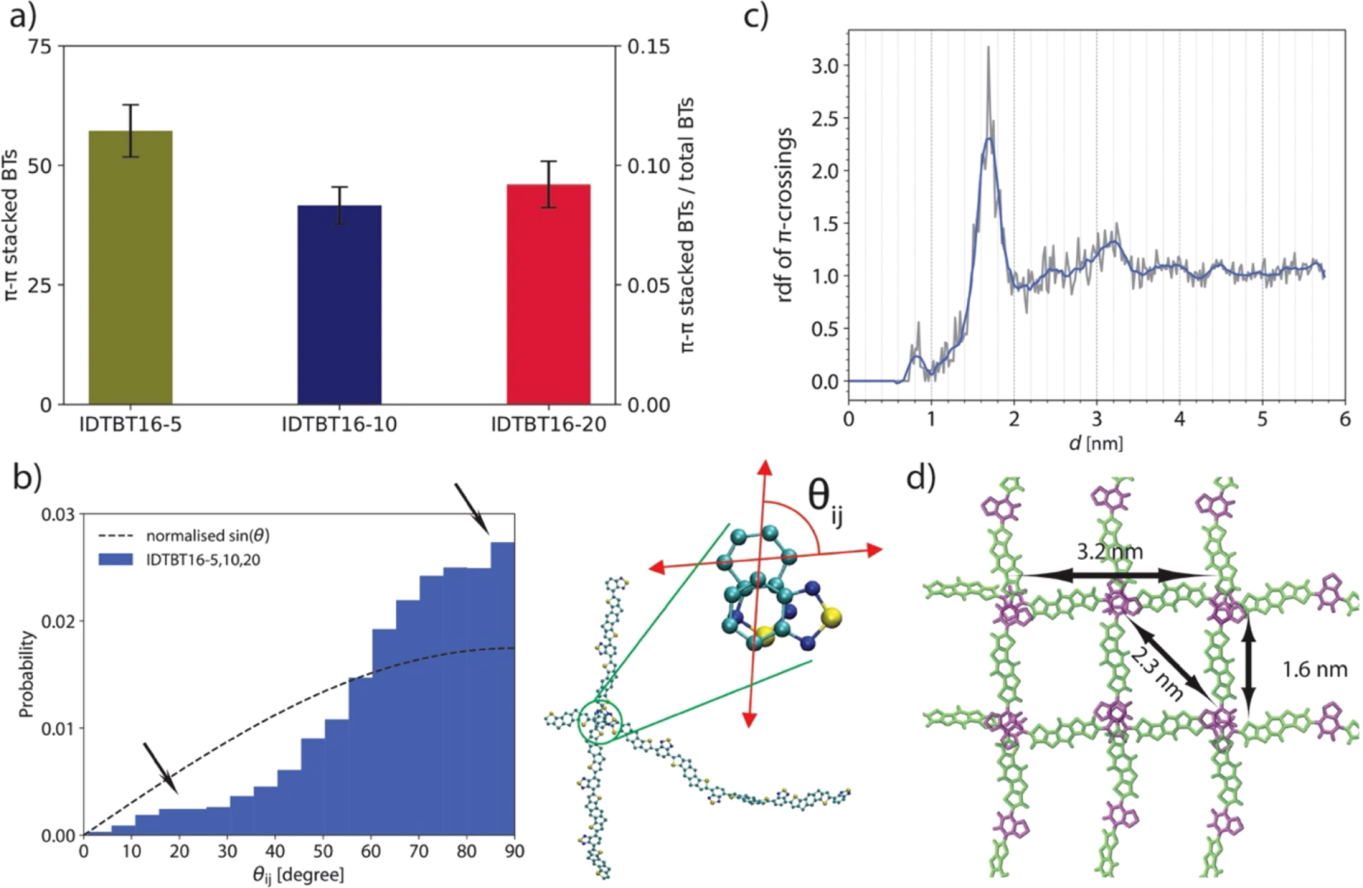
(a) Number of BT-BT π–π interactions for three polymer models. (b) The distribution of relative angle between local director vectors θ_*i*j_ of chains around BT π-crossing junctions through 100 ns of IDTBT16-5, IDTBT16-10, and IDTBT16-20 simulations. (c) rdf of center of mass of BT-BT π-crossings for 100 ns of IDTBT16-5, IDTBT16-10, and IDTBT16-20. (d) Idealized model based on results shown in b and c to illustrate the distances where peaks in the rdf are found and the preferentially perpendicular arrangement of the backbone. Note that the cartoon is excessively idealized and the 3D arrangement of the chains only shows a slight preference to (rather than having an exact) squared lattice structure.

To further analyze the effect of these crossing points on overall morphology, we calculated the radial distribution function (rdf) of the center of mass of BT-BT crossings. Figure 3c shows the collective data for all three polymer models over 100 ns of simulations (four annealing cycles). As shown, a distinct peak appears at 1.7 nm, which is followed by smaller peaks at 2.4 and 3.2 nm. The 1.7 nm is close to the repeating pattern of BT in the polymer chain, with a similar backbone feature shown in the simulated scattering pattern at *q* = 0.4 Å^–1^ (*d* ≈ 1.6 nm). Note that the 0.1 nm difference can be explained by the slightly different position of the center of mass of two BTs belonging to different chains as compared to two adjacent BTs in one chain. 3.2 and 2.4 nm are close to 2*d* and √2*d*, respectively. Also, by pulling out the crossing points contributing to the (small) peak at 0.8 nm, we noticed that it is due to the (rare) π–π interaction between four BT molecules at crossing points so that the average distance between centers of mass of the two BT pair is about 0.8 nm. Note that we observed that >95% of the BT-BT crossings do not show the stacking of more than 2 chains. Accordingly, in Figure 3d, we sketched a 2D cartoon showing an *idealized* model of chain arrangements at crossing points. However, it should be noted that the arrangement of crossing points is three-dimensional and isotropic, and the cartoon only provides a simplified 2D representation of the arrangement.

From the analysis above, BT-BT crossings are clearly the most efficient path for interchain charge transport (as also hypothesized elsewhere^33^) due to the π–π interactions. Therefore, we calculated the chain IDs which share at least one BT-BT crossing and then recognized the cluster of chains in contact by BT-BT crossings for all three polymer models. In Figure 4, the chains belong to each cluster are colored similarly and the individual chains (which do not belong to any cluster) are removed. As shown, by increasing molecular weight, large clusters of polymer chains in contact through BT-BT crossings appear, an observation consistent with the experimental increase of mobility^7,8^ and device performance^34^ with increasing molecular weight. Note that considering the great intrachain transport of IDTBT and considerable orbital overlaps at BT-BT crossings, each color in Figure 4 could represent an efficient path for charge transport.

**Figure 4:**
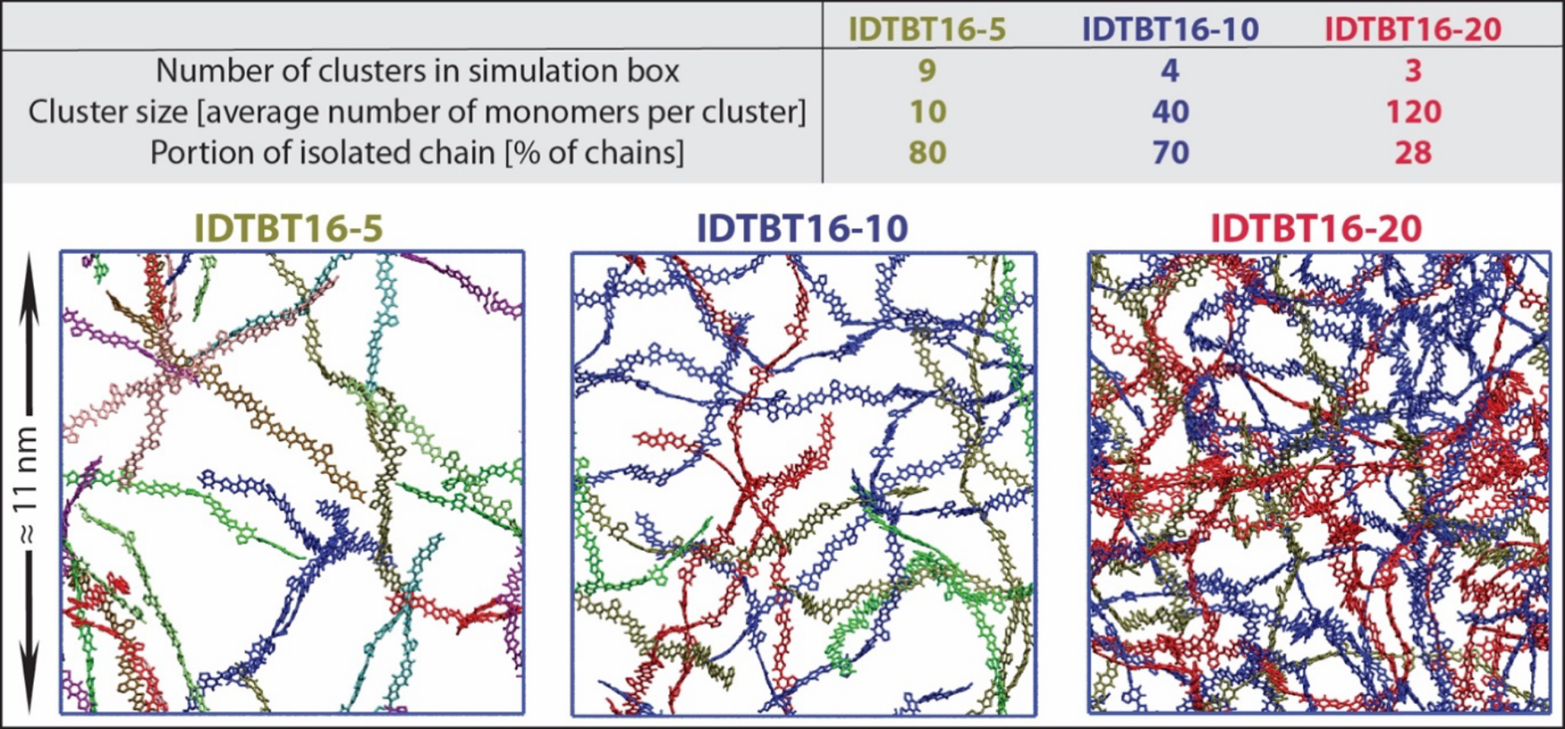
Clusters of chains connected by BT-BT crossings. Each cluster is colored differently, and individual chains are removed from simulation boxes. The statistics of the interconnected clusters of IDTBT chains by BT-BT crossings are listed in the table.

In short, the MD models suggest that IDTBT chains are not packed by the surrounding chains like conventional semicrystalline semiconducting polymers (e.g., P3HT), but BT-BT crossings impose a 3D mesh-like structure which provides efficient sites for interchain connectivity. This feature is unique for IDTBT (and its analogues) and is not shared with other high mobility amorphous-like semiconductors (e.g., those of the DPP family^35,36^), which are demonstrated to form π-π short-range aggregates between chains.

To provide a link between classical simulation and the electronic properties of IDTBT and, by extension, other SCPs, we adopt a method to approximate the bulk electronic structure of a polymer by averaging over the properties of numerous single-chain conformations extracted from MD simulation, with consideration of the chain environment (Figure 5a).^37^ We compute two properties for IDTBT chains: (i) the density of states (DOS) and (ii) the projection on IDT and BT fragments (PDOS). These properties have been used previously to model the electronic structure of SCPs, and one can find detailed descriptions elsewhere (key details are repeated in section 4 of the SI which also gives further information on the sampling procedure).^37,38^ One of the principal findings of our study (Figure 5c) is the near independence in calculation of bulk properties with varying polymer chain length if equivalent sampling is achieved; that is to say, longer chain lengths are not necessary to accurately model the electronic structure of the bulk. We find that the properties calculated with our approach are consistent with those from experiment: the electronic band gap computed to be ∼1.60 eV (see Figure S15 in SI) compares well with the optical band gap of 1.6–1.7 eV determined experimentally.^8^ It is also interesting to note that the valence band here is ∼0.2 eV broader than one obtained from conformations of isolated chains, highlighting the importance of including a realistic description of the environment when computing this key quantity.^38^ Upon calculation of transfer integrals between HOMOs localized on BT fragments at crossing points (Figure 5d), a sizable minority of couplings are larger than |0.1| eV which, in conjunction with non-negligible contribution from BT to the DOS at the band-edge (∼20%) in Figure 5b, confirms the hypothesis that interchain transport is mediated by BT-BT interactions, as already proposed in refs (23 and 28).

**Figure 5:**
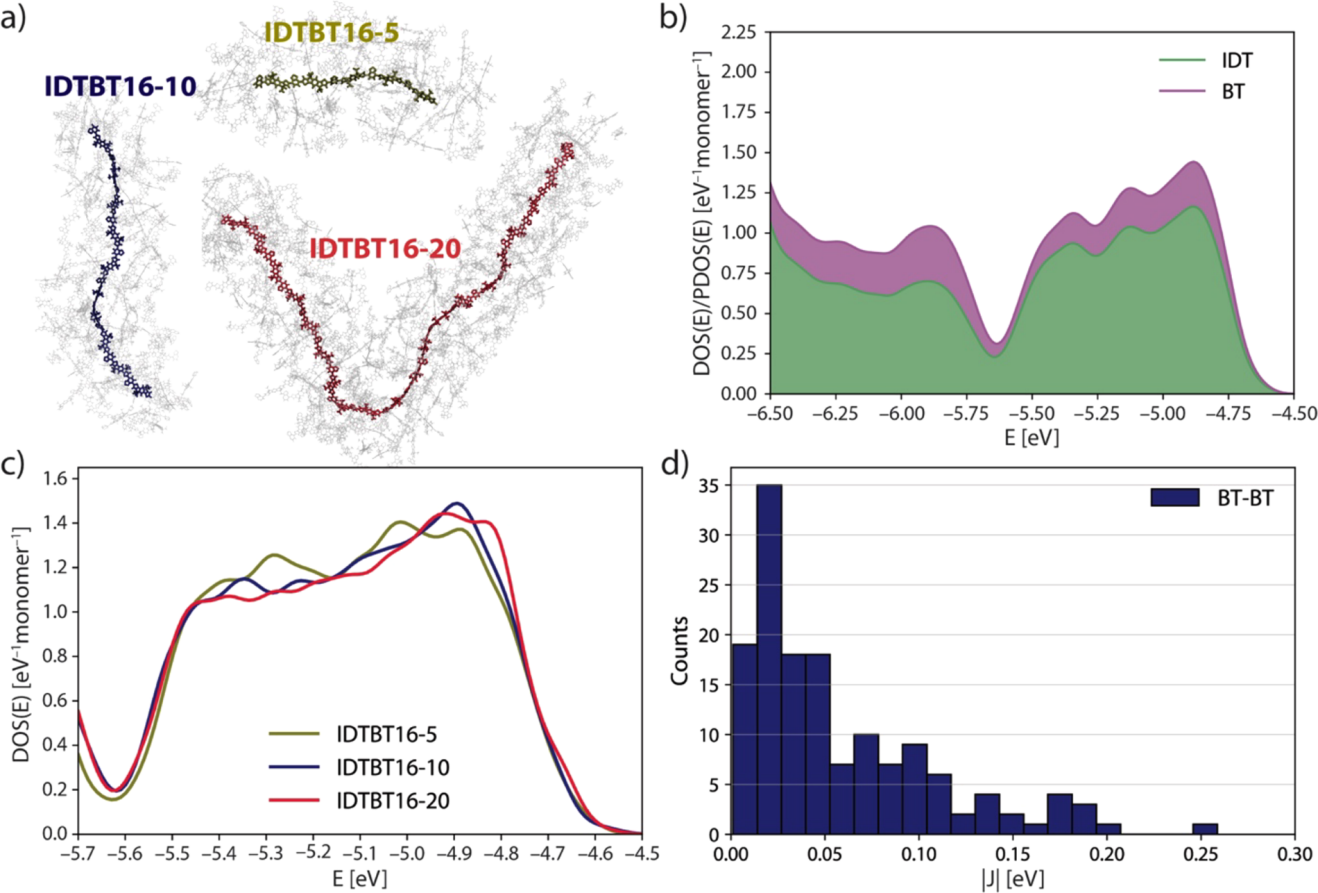
(a) Visualization of central chains and electrostatic cutoffs for electronic structure calculations. (b) PDOS plotted for IDTBT16-10. (c) DOS across a small energy interval of the valence band for IDTBT16-5, IDTBT16-10, and IDTBT16-20. (d) Distribution of HOMO–HOMO transfer integrals calculated for 147 BT-BT crossing points.

In summary, our microscopic models of IDTBT, validated against experimental GIWAXS measurements, predict a noncrystalline structure (i.e., no chain stacking) for IDTBT; however, BT crossings with a relative perpendicular local orientation of chains result in a mesh-like morphology for the polymer. This can reconcile the two (apparently contradictory) observations of “amorphous-like structure”^4^ and existing of a “remarkable short- and medium-range order and unconventional packing”^13^ for IDTBT. Furthermore, these crossing points are shown to be efficient interchain charge transport sites so that for polymers with sufficiently large molecular weights several charge carrier pathways exist. Thus, our models suggest that the extraordinary charge carrier mobility of IDTBT is due to not only the great intrachain charge transport but also the existence of effective interchain transport sites throughout the material (provided by BT-BT π–π interactions) which can significantly contribute to the overall mobility.

## Supporting Information Available

Force field parametrization and model details, simulation and analyses details, X-ray scattering pattern calculation, quantum chemical calculations. (PDF)

## References

[ref1] Noriega R.; Rivnay J.; Vandewal K.; Koch F. P. V.; Stingelin N.; Smith P.; Toney M. F.; Salleo A. A General Relationship between Disorder, Aggregation and Charge Transport in Conjugated Polymers. Nat. Mater. 2013, 12 (11), 1038–1044. 10.1038/nmat3722. 23913173

[ref2] Zhang X.; Bronstein H.; Kronemeijer A. J.; Smith J.; Kim Y.; Kline R. J.; Richter L. J.; Anthopoulos T. D.; Sirringhaus H.; Song K.; Heeney M.; Zhang W.; McCulloch I.; Delongchamp D. M. Molecular Origin of High Field-Effect Mobility in an Indacenodithiophene- Benzothiadiazole Copolymer. Nat. Commun. 2013, 4. 10.1038/ncomms3238. 23900027

[ref3] Ding L.; Yu Z.; Wang X.-Y.; Yao Z.-F.; Lu Y.; Yang C.-Y.; Wang J.-Y.; Pei J. Polymer Semiconductors: Synthesis, Processing, and Applications. Chem. Rev. 2023, 123 (12), 7421–7497. 10.1021/acs.chemrev.2c00696. 37232480

[ref4] Venkateshvaran D.; Nikolka M.; Sadhanala A.; Lemaur V.; Zelazny M.; Kepa M.; Hurhangee M.; Kronemeijer A. J.; Pecunia V.; Nasrallah I.; Romanov I.; Broch K.; McCulloch I.; Emin D.; Olivier Y.; Cornil J.; Beljonne D.; Sirringhaus H. Approaching Disorder-Free Transport in High-Mobility Conjugated Polymers. Nature 2014, 515 (7527), 384–388. 10.1038/nature13854. 25383522

[ref5] Zhang W.; Smith J.; Watkins S. E.; Gysel R.; McGehee M.; Salleo A.; Kirkpatrick J.; Ashraf S.; Anthopoulos T.; Heeney M.; McCulloch I. Indacenodithiophene Semiconducting Polymers for High-Performance, Air-Stable Transistors. J. Am. Chem. Soc. 2010, 132 (33), 11437–11439. 10.1021/ja1049324. 20677750

[ref6] Kim S. H.; Yook H.; Sung W.; Choi J.; Lim H.; Chung S.; Han J. W.; Cho K. Extremely Suppressed Energetic Disorder in a Chemically Doped Conjugated Polymer. Adv. Mater. 2023, 35 (1), 1–11. 10.1002/adma.202207320. 36271732

[ref7] Zhao B.; Pei D.; Jiang Y.; Wang Z.; An C.; Deng Y.; Ma Z.; Han Y.; Geng Y. Simultaneous Enhancement of Stretchability, Strength, and Mobility in Ultrahigh-Molecular-Weight Poly(Indacenodithiophene- Co-Benzothiadiazole). Macromolecules 2021, 54 (21), 9896–9905. 10.1021/acs.macromol.1c01513.

[ref8] Wadsworth A.; Chen H.; Thorley K. J.; Cendra C.; Nikolka M.; Bristow H.; Moser M.; Salleo A.; Anthopoulos T. D.; Sirringhaus H.; McCulloch I. Modification of Indacenodithiophene-Based Polymers and Its Impact on Charge Carrier Mobility in Organic Thin-Film Transistors. J. Am. Chem. Soc. 2020, 142 (2), 652–664. 10.1021/jacs.9b09374. 31851506

[ref9] Zheng Y.; Wang G. J. N.; Kang J.; Nikolka M.; Wu H. C.; Tran H.; Zhang S.; Yan H.; Chen H.; Yuen P. Y.; Mun J.; Dauskardt R. H.; McCulloch I.; Tok J. B. H.; Gu X.; Bao Z. An Intrinsically Stretchable High-Performance Polymer Semiconductor with Low Crystallinity. Adv. Funct. Mater. 2019, 29 (46), 1905340 10.1002/adfm.201905340.

[ref10] Sommerville P. J. W.; Balzer A. H.; Lecroy G.; Guio L.; Wang Y.; Onorato J. W.; Kukhta N. A.; Gu X.; Salleo A.; Stingelin N.; Luscombe C. K. Influence of Side Chain Interdigitation on Strain and Charge Mobility of Planar Indacenodithiophene Copolymers. ACS Polym. Au 2023, 3 (1), 59–69. 10.1021/acspolymersau.2c00034. 36785836 PMC9912480

[ref11] Lemaur V.; Cornil J.; Lazzaroni R.; Sirringhaus H.; Beljonne D.; Olivier Y. Resilience to Conformational Fluctuations Controls Energetic Disorder in Conjugated Polymer Materials: Insights from Atomistic Simulations. Chem. Mater. 2019, 31 (17), 6889–6899. 10.1021/acs.chemmater.9b01286.

[ref12] Cao X.; Li H.; Hu J.; Tian H.; Han Y.; Meng B.; Liu J.; Wang L. An Amorphous N-Type Conjugated Polymer with an Ultra-Rigid Planar Backbone. Angew. Chem. 2023, 135 (2), 1–7. 10.1002/ange.202212979. 36345132

[ref13] Cendra C.; Balhorn L.; Zhang W.; O’Hara K.; Bruening K.; Tassone C. J.; Steinrück H. G.; Liang M.; Toney M. F.; McCulloch I.; Chabinyc M. L.; Salleo A.; Takacs C. J. Unraveling the Unconventional Order of a High-Mobility Indacenodithiophene-Benzothiadiazole Copolymer. ACS Macro Lett. 2021, 10 (10), 1306–1314. 10.1021/acsmacrolett.1c00547. 35549036

[ref14] Chen H.; Wadsworth A.; Ma C.; Nanni A.; Zhang W.; Nikolka M.; Luci A. M. T.; Perdigão L. M. A.; Thorley K. J.; Cendra C.; Larson B.; Rumbles G.; Anthopoulos T. D.; Salleo A.; Costantini G.; Sirringhaus H.; McCulloch I. The Effect of Ring Expansion in Thienobenzo[ b]Indacenodithiophene Polymers for Organic Field-Effect Transistors. J. Am. Chem. Soc. 2019, 141 (47), 18806–18813. 10.1021/jacs.9b09367. 31613619

[ref15] Nikolka M.; Broch K.; Armitage J.; Hanifi D.; Nowack P. J.; Venkateshvaran D.; Sadhanala A.; Saska J.; Mascal M.; Jung S. H.; Lee J. K.; McCulloch I.; Salleo A.; Sirringhaus H. High-Mobility, Trap-Free Charge Transport in Conjugated Polymer Diodes. Nat. Commun. 2019, 10 (1), 1–9. 10.1038/s41467-019-10188-y. 31073179 PMC6509340

[ref16] Rühle V.; Kirkpatrick J.; Andrienko D. A Multiscale Description of Charge Transport in Conjugated Oligomers. J. Chem. Phys. 2010, 132 (13), 134103 10.1063/1.3352568. 20387917

[ref17] Melnyk A.; Junk M. J. N.; McGehee M. D.; Chmelka B. F.; Hansen M. R.; Andrienko D. Macroscopic Structural Compositions of π-Conjugated Polymers: Combined Insights from Solid-State NMR and Molecular Dynamics Simulations. J. Phys. Chem. Lett. 2017, 8 (17), 4155–4160. 10.1021/acs.jpclett.7b01443. 28809493

[ref18] Rezayani M.; Sharif F.; Makki H. Understanding Ion Diffusion in Anion Exchange Membranes; Effects of Morphology and Mobility of Pendant Cationic Groups. J. Mater. Chem. A 2022, 10 (35), 18295–18307. 10.1039/D2TA04400E.

[ref19] Rezayani M.; Sharif F.; Makki H. Role of Side-Chain Lengths on Hydronium Mobility in Sulfonated Poly(Ether Sulfone) Proton-Conducting Model Membranes. J. Phys. Chem. C 2023, 127 (18), 8462–8472. 10.1021/acs.jpcc.3c01026.

[ref20] Landi A.; Reisjalali M.; Elliott J. D.; Matta M.; Carbone P.; Troisi A. Simulation of Polymeric Mixed Ionic and Electronic Conductors with a Combined Classical and Quantum Mechanical Model. J. Mater. Chem. C 2023, 11, 8062–8073. 10.1039/D2TC05103F. PMC10286221 37362027

[ref21] Reisjalali M.; Manurung R.; Carbone P.; Troisi A. Development of Hybrid Coarse-Grained Atomistic Models for Rapid Assessment of Local Structuring of Polymeric Semiconductors. Mol. Syst. Des. Eng. 2022, 7 (3), 294–305. 10.1039/D1ME00165E. 35646391 PMC9074845

[ref22] Guilbert A. A. Y.; Zbiri M.; Dunbar A. D. F.; Nelson J. Quantitative Analysis of the Molecular Dynamics of P3HT:PCBM Bulk Heterojunction. J. Phys. Chem. B 2017, 121 (38), 9073–9080. 10.1021/acs.jpcb.7b08312. 28834430

[ref23] Guilbert A. A. Y.; Urbina A.; Abad J.; Díaz-Paniagua C.; Batallán F.; Seydel T.; Zbiri M.; García-Sakai V.; Nelson J. Temperature-Dependent Dynamics of Polyalkylthiophene Conjugated Polymers: A Combined Neutron Scattering and Simulation Study. Chem. Mater. 2015, 27 (22), 7652–7661. 10.1021/acs.chemmater.5b03001.

[ref24] Dobryden I.; Korolkov V. V.; Lemaur V.; Waldrip M.; Un H. I.; Simatos D.; Spalek L. J.; Jurchescu O. D.; Olivier Y.; Claesson P. M.; Venkateshvaran D. Dynamic Self-Stabilization in the Electronic and Nanomechanical Properties of an Organic Polymer Semiconductor. Nat. Commun. 2022, 13 (1), 1–11. 10.1038/s41467-022-30801-x. 35654891 PMC9163058

[ref25] Ponder J. F.; Chen H.; Luci A. M. T.; Moro S.; Turano M.; Hobson A. L.; Collier G. S.; Perdigão L. M. A.; Moser M.; Zhang W.; Costantini G.; Reynolds J. R.; McCulloch I. Low-Defect, High Molecular Weight Indacenodithiophene (IDT) Polymers Via a C-H Activation: Evaluation of a Simpler and Greener Approach to Organic Electronic Materials. ACS Mater. Lett. 2021, 3 (10), 1503–1512. 10.1021/acsmaterialslett.1c00478.

[ref26] Keene S. T.; Michaels W.; Melianas A.; Quill T. J.; Fuller E. J.; Giovannitti A.; McCulloch I.; Talin A. A.; Tassone C. J.; Qin J.; Troisi A.; Salleo A. Efficient Electronic Tunneling Governs Transport in Conducting Polymer-Insulator Blends. J. Am. Chem. Soc. 2022, 144 (23), 10368–10376. 10.1021/jacs.2c02139. 35658455 PMC9204759

[ref27] Makki H.; Troisi A. Morphology of Conducting Polymer Blends at the Interface of Conducting and Insulating Phases: Insight from PEDOT:PSS Atomistic Simulations. J. Mater. Chem. C 2022, 10 (42), 16126–16137. 10.1039/D2TC03158B. PMC9632246 36387833

[ref28] Lehn J. M.; Gölitz P. Concepts in Biophysical Chemistry. Chem. - A Eur. J. 1996, 2 (7), 751 10.1002/chem.19960020704.

[ref29] Heil C. M.; Ma Y.; Bharti B.; Jayaraman A. Computational Reverse-Engineering Analysis for Scattering Experiments for Form Factor and Structure Factor Determination (“P(q) and S(q) CREASE”). JACS Au 2023, 3 (3), 889–904. 10.1021/jacsau.2c00697. 37006757 PMC10052275

[ref30] Alessandri R.; Uusitalo J. J.; De Vries A. H.; Havenith R. W. A.; Marrink S. J. Bulk Heterojunction Morphologies with Atomistic Resolution from Coarse-Grain Solvent Evaporation Simulations. J. Am. Chem. Soc. 2017, 139 (10), 3697–3705. 10.1021/jacs.6b11717. 28209056 PMC5355903

[ref31] Siemons N.; Pearce D.; Cendra C.; Yu H.; Tuladhar S. M.; Hallani R. K.; Sheelamanthula R.; LeCroy G. S.; Siemons L.; White A. J. P.; McCulloch I.; Salleo A.; Frost J. M.; Giovannitti A.; Nelson J. Impact of Side-Chain Hydrophilicity on Packing, Swelling, and Ion Interactions in Oxy-Bithiophene Semiconductors. Adv. Mater. 2022, 34 (39), 1–9. 10.1002/adma.202204258. 35946142

[ref32] Thomas T. H.; Harkin D. J.; Gillett A. J.; Lemaur V.; Nikolka M.; Sadhanala A.; Richter J. M.; Armitage J.; Chen H.; McCulloch I.; Menke S. M.; Olivier Y.; Beljonne D.; Sirringhaus H. Short Contacts between Chains Enhancing Luminescence Quantum Yields and Carrier Mobilities in Conjugated Copolymers. Nat. Commun. 2019, 10 ( (1), ). 10.1038/s41467-019-10277-y. PMC6565747 31197152

[ref33] Jacobs I. E.; D’avino G.; Lemaur V.; Lin Y.; Huang Y.; Chen C.; Harrelson T. F.; Wood W.; Spalek L. J.; Mustafa T.; O’keefe C. A.; Ren X.; Simatos D.; Tjhe D.; Statz M.; Strzalka J. W.; Lee J. K.; Mcculloch I.; Fratini S.; Beljonne D.; Sirringhaus H. Structural and Dynamic Disorder, Not Ionic Trapping, Controls Charge Transport in Highly Doped Conducting Polymers. J. Am. Chem. Soc. 2022, 144 (7), 3005–3019. 10.1021/jacs.1c10651. 35157800 PMC8874922

[ref34] Ashraf R. S.; Schroeder B. C.; Bronstein H. A.; Huang Z.; Thomas S.; Kline R. J.; Brabec C. J.; Rannou P.; Anthopoulos T. D.; Durrant J. R.; McCulloch I. The Influence of Polymer Purification on Photovoltaic Device Performance of a Series of Indacenodithiophene Donor Polymers. Adv. Mater. 2013, 25 (14), 2029–2034. 10.1002/adma.201300027. 23417853

[ref35] Yiu A. T.; Beaujuge P. M.; Lee O. P.; Woo C. H.; Toney M. F.; Fréchet J. M. J. Side-Chain Tunability of Furan-Containing Low-Band-Gap Polymers Provides Control of Structural Order in Efficient Solar Cells. J. Am. Chem. Soc. 2012, 134 (4), 2180–2185. 10.1021/ja2089662. 22191680

[ref36] Reisjalali M.; Burgos-Mármol J. J.; Manurung R.; Troisi A. Local Structuring of Diketopyrrolopyrrole (DPP)-Based Oligomers from Molecular Dynamics Simulations. Phys. Chem. Chem. Phys. 2021, 23 (35), 19693–19707. 10.1039/D1CP03257G. 34525153

[ref37] Qin T.; Troisi A. Relation between Structure and Electronic Properties of Amorphous MEH-PPV Polymers. J. Am. Chem. Soc. 2013, 135 (30), 11247–11256. 10.1021/ja404385y. 23829780

[ref38] Manurung R.; Li P.; Troisi A. Rapid Method for Calculating the Conformationally Averaged Electronic Structure of Conjugated Polymers. J. Phys. Chem. B 2021, 125 (23), 6338–6348. 10.1021/acs.jpcb.1c02866. 34097424

