# Supplementary material for: Microstructural Model of Indacenodithiophene-co-benzothiadiazole Polymer: π-Crossing Interactions and Their Potential Impact on Charge Transport
Source: J Phys Chem Lett. 2023 Sep 27;14(39):8867–73. doi: 10.1021/acs.jpclett.3c02305 (PMC10561260; doi:10.1021/acs.jpclett.3c02305)
Supplement: Supplementary file 1 — jz3c02305_si_001.pdf [file jz3c02305_si_001.pdf]

Supporting Information For:

Microstructural Model of Indacenodithiophene-co-benzothiadiazole Polymer: π-crossing Interactions and Their Potential Impact on Charge Transport

Hesam Makki[1*], Colm Burke[1], Alessandro Troisi[1*]

[1]Department of Chemistry and Materials Innovation Factory, University of Liverpool, Liverpool L69 7ZD, U.K.

Corresponding Authors:
Hesam Makki
Email: h.makki@liverpool.ac.uk
Alessandro Troisi
Email: a.troisi@liverpool.ac.uk

S1. Force Field Parametrisation and Model Details

Force field parameters of the backbone and sidechains of the polymer were obtained separately. OPLS force field was used for bonded and non-bonded parameters of the side chains. The backbone parameters were derived from optimised structure of IDT-BT monomer with two methyl groups attached to each sidechain connection point of IDT (in total four methyl groups representing the place of four side chains that will be attached later) and the monomer was capped with IDT and BT fragments (see Figure S1, the capping fragments are marked with blue and red ellipses). Note that all DFT calculations were performed by Gaussian 16 by using B3lyp hybrid functional and cc-pVTZ basis set for all steps of force field calculations.

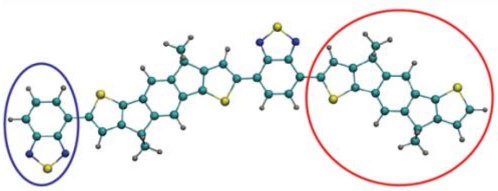

Figure S1. IDT-BT monomer structure. The blue (BT) and red (IDT) highlighted fragments were used to cap the repeat unit.

The monomer optimisation process is summarised in “monomer optimisation”. The backbone non-bonded and bonded parameterisation methods are detailed in “non-bonded parameters” and “bonded parameters”, respectively. The sidechain attachment procedure and the backbone-sidechain connection point parametrisation are explained in section “sidechain attachment procedure”. “Polymer models” summarises the resulting IDT-BT polymers.

Monomer Optimisation

For the model monomer shown in Figure S1, all possible *Cis-Trans* isomers have been made (see Figure S2) and their total energies of optimised structures were calculated and the lowest energy structure (7) was used as a starting point for the other component of the force field generation.

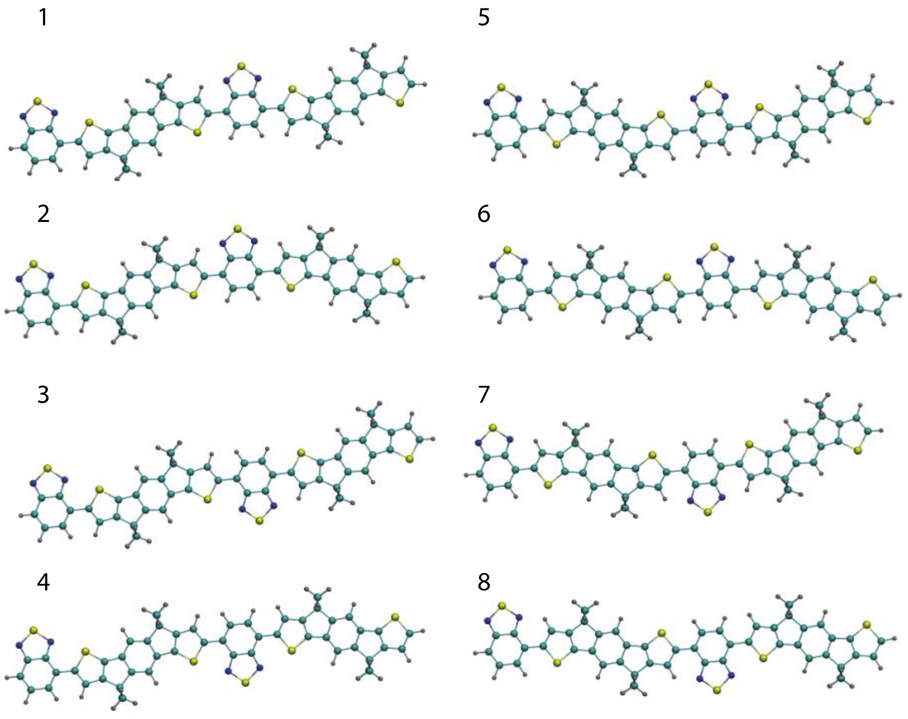

Figure S2. All possible Cis-Trans isomers of IDT-BT. Structure 7 shows the minimum energy value among all.

Non-bonded Parameters

Lennard Jones parameters

The Lennard jones parameters of atoms in the polymer backbone were directly taken from their analogues in OPLS force field.

Atomic Charge of Backbone (Conjugated Part)

The atomic charges were calculated by using CHELPG scheme (developed by Breneman and Wiberg, J. Comp. Chem. 1990, 11, 361) on the optimised structure of the monomer shown in Figure S1. The resulting point charges from CHELPG computation for one repeat unit (RU, i.e., the monomer structure after removing the capping (BT and IDT) fragments, see Figure S3) are shown in table S1. The sum of point charges for the whole monomer was zero and, after removing the capping BT and IDT fragments, the total charge of one RU was calculated –0.004 e. The extra charge of the RU was redistributed on all atoms (equally) so that the total charge of each RU in the polymers equals to zero.

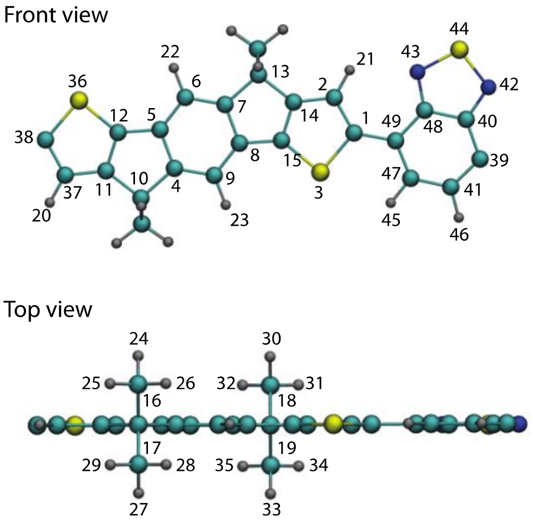

Figure 3. Atom labels as used for force field parameterisation of one IDT-BT repeat unit (RU).

Table S1. Atomic charges as calculated by CHELPG method and as used for the repeat unit force field (RU-FF). Note that the total charge of each repeat unit is set to zero by redistribution of the total excess charge calculated by CHELPG.

Atom	CHELPG	RU-FF	Atom	CHELPG	RU-FF	Atom	CHELPG	RU-FF
1	0.0271	0.027	18	–0.1733	–0.173	35	0.0484	0.048
2	–0.1135	–0.113	19	–0.1733	–0.173	36	–0.1246	–0.124
3	–0.1202	–0.12	20	0.0721	0.072	37	–0.0898	–0.089
4	–0.153	–0.153	21	0.0692	0.069	38	0.0154	0.015
5	0.1411	0.141	22	0.1694	0.169	39	–0.0258	–0.025
6	–0.2122	–0.212	23	0.1742	0.174	40	0.2328	0.233
7	–0.1363	–0.136	24	0.0072	0.007	41	–0.1263	–0.126
8	0.1274	0.127	25	0.0422	0.042	42	–0.3157	–0.315
9	–0.2110	–0.21	26	0.0146	0.014	43	–0.3115	–0.311
10	0.4616	0.461	27	0.0072	0.007	44	0.2580	0.258
11	–0.1156	–0.115	28	0.0146	0.014	45	0.1437	0.144
12	–0.0012	–0.001	29	0.0422	0.042	46	0.1384	0.138
13	0.4413	0.441	30	0.0097	0.01	47	0.2141	0.214
14	–0.0813	–0.081	31	0.0484	0.048	48	–0.1584	–0.158
15	–0.0100	–0.009	32	0.0204	0.02	49	–0.0039	–0.003
16	–0.1591	–0.159	33	0.0098	0.01	**SUM**	**–0.0040**	**0.000**
17	–0.1591	–0.159	34	0.0204	0.02			

Bonded Parameters

Bonds and Angles

Harmonic potentials in the forms of equations SE1 and SE2 were used for implementing bond and angle interactions, respectively, in the force field.

$$V_b(r_{ij}) = \frac{1}{2} k_{ij}^{b} (r_{ij}-b_{ij})^2 \qquad \text{(SE1)}$$

$$V_a(\theta_{ijk}) = \frac{1}{2} k_{ijk}^{\theta} (\theta_{ijk} - \theta_{ijk}{}^{0})^2 \qquad \text{(SE2)}$$

where V_b and V_a are bond and angle potentials, k^b and K^θ are the force constants representing the stiffness of the bond and angle, and b_{ij} and θ_{ijk} are the bond and angle equilibrium values taken from DFT-optimised monomer for each combination of bonded atoms. It is important that the distances and angles are consistent with DFT calculations because the MD simulations will be used to generate models for DFT calculations. Note that employing force field like GAFF that ignore the pi-bonding may introduce errors.

The stiffness of all bonds (k_{ij}^{b}) and angles (k_{ijk}^{θ}) were set to 320,000.0 kJ/mol/nm^2 and 500.0 kJ/mol/rad^2.

Intra Fragment Torsional Potentials

The torsional potentials for internal dihedral angles of IDT and BT fragments in the RU were implemented in form of a Ryckaert-Bellemans function (equation SE3) for all dihedral angles around sp2 hybridised heavy atoms. These fragments are flat and rigid due to their (partial) double bond nature. A representative and well-studied example for a flat and sp2 hybridised molecule is the benzene ring; thus, the OPLS constants of Ryckaert-Bellemans function for carbon atoms of benzene molecule was used for these dihedral potentials. It is worth noting that benzene is an ideal molecule if one wants a single torsional potential for conjugated fragments as each bond is 50% double and 50% single. The Ryckaert-Bellemans reads as:

$$V_{rb}(\phi_{ijkl}) = \sum_{n=0}^{5} C_n(\cos(\psi))^n \qquad \text{SE3}$$

where V_{rb} is the torsional potential of the dihedral angle between the planes of ijk and jkl atoms. Note that $\psi=\phi$-180 and C_n are the six constants of the function. The C_n [kJ/mol] values of the C-C-C-C dihedral of the benzene ring from OPLS force field are shown in Table 2.

Table 2. The OPLS force field constants of Ryckaert-Bellemans function for C-C-C-C dihedral angle of benzene.

C_0	C_1	C_2	C_3	C_4	C_5
30.334	0.0	-30.334	0	0	0

For the dihedral angles in which the sp3 hybridised carbons (the connecting carbon to the sidechain) take part, equation S4 was used (proper dihedral type 1 in GROMACS). Note that all equilibrium dihedral angle values (ϕ_s in equation S4) were directly taken from the optimised monomer.

$$V_d(\phi_{ijkl}) = k_\phi(1+\cos(\phi-\phi_s)) \qquad \text{SE4}$$

k_ϕ of 10 kJ/mol was used for all dihedrals around the sp3 carbons connecting the backbone to the sidechains. Note that ϕ= 0 is corresponding to the cis configuration (i.e., *ijk* and *jkl* on the same side).

Inter Fragment Torsional Potential (Connecting Point between IDT and BT)

Torsional potential (V^{DFT}) of dihedral angle between IDT and BT ($\phi_{IDT\text{-}BT}$) in the monomer structure (marked with red circles in Figure S4 a) was calculated by DFT (via B3LYP/cc-pvtz) through a dihedral scan with 10° spacing (in total 37 scans from –180° to 180°). Figure S4 b

shows the calculated torsional potential as a function of dihedral angle. It should be noted that V^{DFT} shows the total potential energy of the monomer structure at each of 37 dihedral angle points. Therefore, after implementing this torsional potential correctly as a force field parameter, the total potential energy of the monomer at each dihedral angle as calculated by the force field should match the $V^{DFT}(\phi_{IDT\text{-}BT})$. To this end, we used the parametrisation scheme as explained below.

The DFT-optimised structures at each $\phi_{IDT\text{-}BT}$ (i.e., –180, –170, …, 170, 180) were obtained and used as the input coordinate file. Then, for each structure, an energy minimisation based on steepest descent algorithm using the generated force field parameters (excluding the torsional potential of $\phi_{IDT\text{-}BT}$) with a stiff dihedral restraint (30,000 kJ/mol/rad), which ensures $\phi_{IDT\text{-}BT}$ remains reasonably constant (< 1° fluctuations) during minimisation, was performed. The total energy (V^{FF}), excluding the energies of the restrained dihedral, for each structure (in total 37 values) were calculated after energy minimisation.

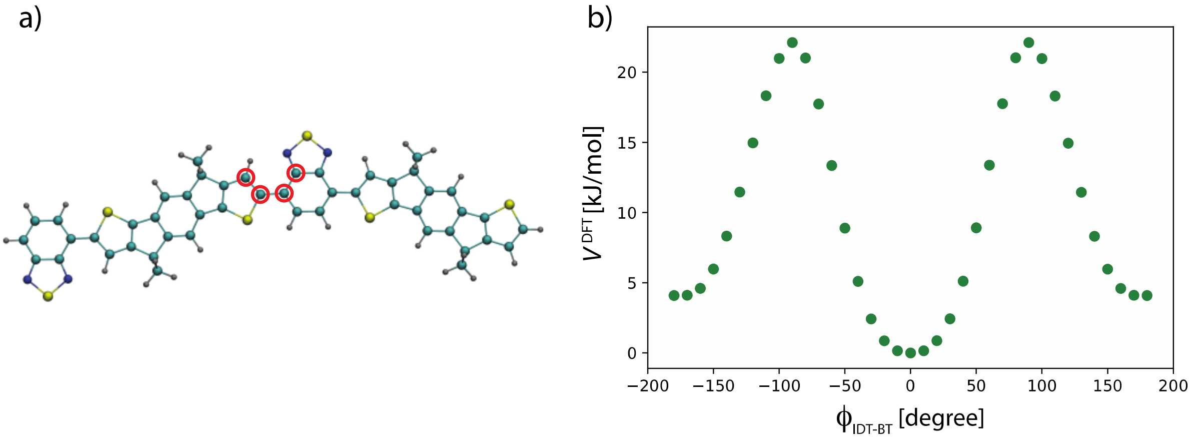

Figure S4. (a) IDT-BT monomer capped with BT (left-end) and IDT (fight-end) molecules; the red circle shows the place of rotation for dihedral scan ($\phi_{IDT\text{-}BT}$). (b) DFT calculated torsional potential by B3LYP/cc-pvtz.

Figure S5 a shows V^{FF} values for all 37 structures. Accordingly, the torsional potential for the force field will be $V^{CORR} = V^{DFT} - V^{FF}$. Figure S5 b shows the correct torsional potential, as it is given to the force field in the format of a table potential for MD simulations. The dihedral table was provided as a three-column table. The first column shows angles (-180 to and including 180, 1-degree spacing is recommended), the second column is the potential value (a cubic spline was fitted on 10-degree spline and the potential for every degree was calculated), and the third column represents the negative value of the first derivates of the potential (i.e., force) as obtained from the cubic spline fit.

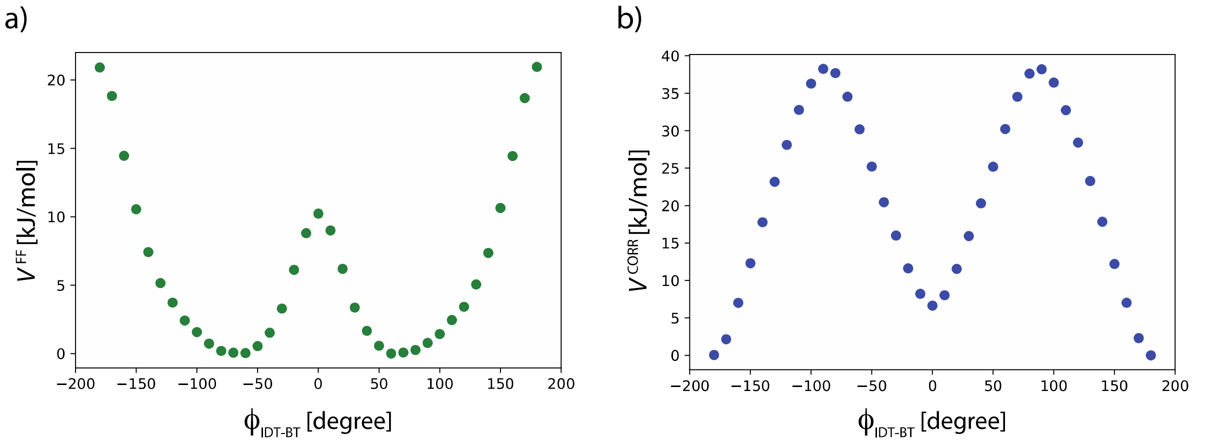

Figure S5. (a) Total energy (excluding the restraint energy imposed to keep $\phi_{IDT\text{-}BT}$ constant) based on the force field (excluding the $\phi_{IDT\text{-}BT}$ torsional potential) for energy minimised structures. (b) Force field torsional potential ($V^{CORR} = V^{DFT} - V^{FF}$).

As a final check, we calculated the total energy of the monomer, but this time the force filed torsional potential (V^{CORR}) was also included. Figures S6 shows the comparison between the total energy (excluding the dihedral restraint energy) from force field (V^{tot}) and the DFT calculated torsional potential (V^{DFT}) for IDT-BT. As shown, the total potential energy of the monomer after imposing the implemented torsional potential (V^{CORR}) is matching the DFT calculated values (V^{DFT}).

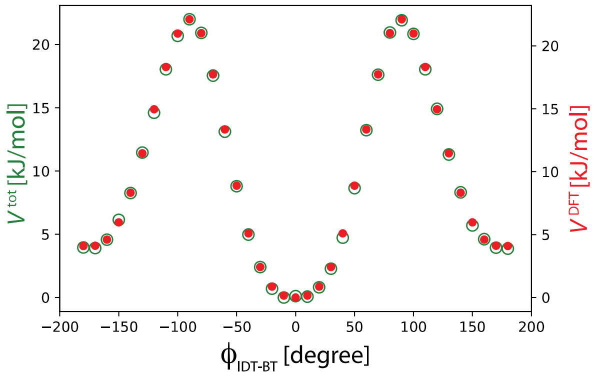

Figure S6. Comparison between DFT calculated (V^{DFT}) and force field calculated (V^{tot}) total potential energy at each dihedral angle of ϕ_{IDT-BT}.

Sidechain Attachment Procedure

The optimised RU structure (coordinates) and force field parameters have been obtained so far. The next step is to attach side chains to the repeat unit and add the force field parameters accordingly. As earlier mentioned, OPLS force field parameters were used for sidechains. However, the interactions between the backbone and the sidechain at the connection points should be treated correctly.

First, the sidechains are assumed to have a total zero net charge. Each methylene ($-CH_2-$) and the end methyl (CH_3) group for each sidechain are parametrised by the united atom parameters of OPLS. Therefore, the total charge of each united atom (and accordingly, the charge of each sidechain) is zero. However, to attach a side chain to each of four methyl groups in the RU, one hydrogen atom of the methyl group should be removed, and its charge should be redistributed on the remaining two hydrogen atoms, see Figure S7. In this way, the total charge of the repeat unit with sidechain (RU-SC) will remain zero.

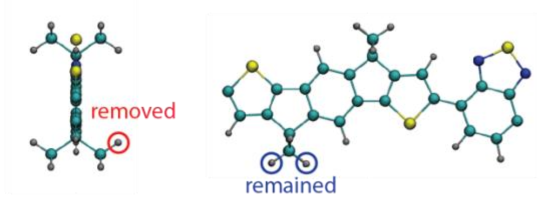

Figure S7. One hydrogen of each methyl group attached to IDT is removed (red circle) and its charge is redistributed on the two remaining hydrogens (blue circles).

The bonded potentials defined around the connection points also need a reasonable treatment. Figure S8 shows the RU-SC structure. The atom labeling around one of the two connection points are shown. The sp2 hybridised carbon of IDT are shown in green numbers, the sp3 carbon and hydrogen atoms are represented by blue numbers, and the sidechain united atoms are marked with red numbers.

After removing one hydrogen of each methyl group, all the bonded parameters in which this hydrogen was involved were removed from the force field. Then the new bonded potentials were added to the force field as explained here. Based on the atom labeling shown in Figure S8, the bond potential for 16-24 bond was taken from OPLS and added to the force field. Also,

all angle potentials for any newly formed angle in which any sidechain united atom exists (e.g., 51-24-16, 24-16-26, 24-16-25, 24-16-10, etc.) were taken from OPLS and added to the force field. In a same way, the potential of all the newly formed dihedrals in which any sidechain united atom exists (e.g., 51-24-16-26, 24-16-10-17, etc.) excluding the ones that sp2 hybridised carbons of the IDT also exist (i.e., 24-16-10-11, and 24-16-10-4) were taken from OPLS and added to the force field. Excluding the two dihedral potentials for each sidechain in which one sidechain united atom (e.g., 24) and one sp2 hybridised carbon atom (e.g., 4 and 11) take part is due to avoiding any distortion in the flatness of the conjugated fragment (IDT) by imposing additional dihedral potentials on it.

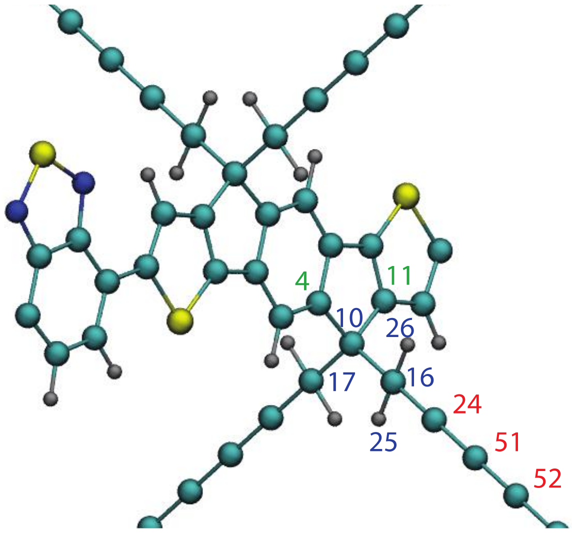

Figure S8. Atom labeling of the RU-SC (repeat unit with side chain). Green, blue, and red colored numbers represent sp2 carbons, non-sp2 hybridised atoms, and sidechain united atoms, respectively.

In this way, we constructed RU-SC structures with different side chain lengths. RU-SC16, RU-SC8, RU-SC4, and RU-SC1 are shown in figure S9. For instance, RU-SC8 is the repeat unit of IDT-BT polymer with four side chains with a length of 8 carbon atoms.

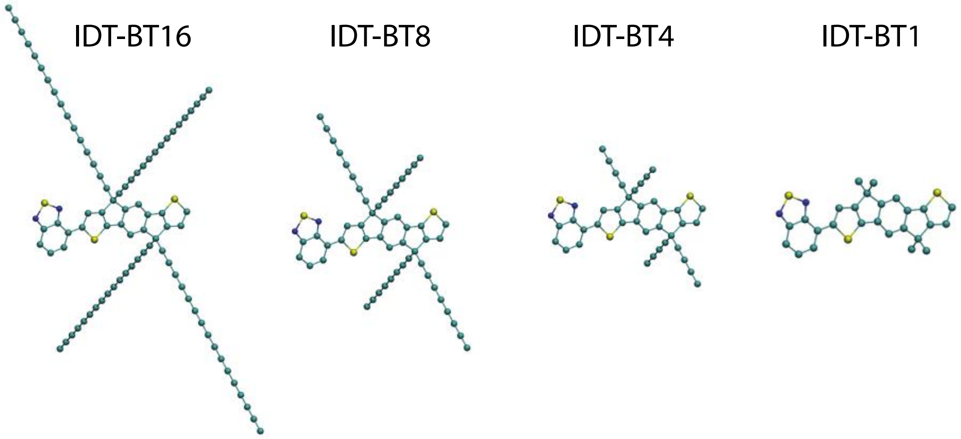

Figure S9. IDT-BT repeat units with different sidechain lengths.

Polymer models

For all RU-SC structures as shown in Figure S9, a polymer with *DP* (degree of polymerisation) =5 was made, i.e., IDT-BT16-5, IDT-BT8-5, IDT-BT4-5, and IDT-BT1-5. For IDT-BT16, polymers with *DP*=10 and 20 were also made, i.e., IDT-BT16-10 and IDT-BT16-20. The force field of polymers are simply made by repeating the force field of each RU-SC. Note that all polymers were capped with one united atom carbon (with zero charge) at both ends and improper dihedral potentials were used to keep it coplanar with the backbone of the polymer.

S2. Simulation and Analyses Details

Simulation Procedure and Parameters

Fully stretched IDTBT chains were randomly inserted into the simulation box and after energy minimisation a rapid contraction of the box were done (under NPT and with P = 1000 bar) to quickly pack the box to the correct density. Then several annealing cycles, as shown in the main manuscript, were performed to relax the structures. Relaxation simulations were performed under NPT condition with time step of 3 fs by using GROMACS. A 1.0 nm cutoff for Lennard Johns and electrostatic interactions was used and all nonbonded interactions for 1-2 and 1-3 bonded pairs were excluded and a scaling factor of 0.5 was used for 1-4 bonded pairs. V-rescale thermostat and C-rescale barostat were used for packing steps and Nose-Hoover thermostat and Parrinello-Rahman barostat were employed for equilibration runs. Verlet cut-off scheme was employed for non-bonded interactions and Particle-mesh Ewald was used for long-range electrostatic interactions. Examples of coordinate, topology, and run files can be found in https://github.com/HMakkiMD/IDTBT.

T_g evaluation and annealing procedure setup

IDTBT shows a glass transition temperature T_g of around 510 K in our MD simulations (see Figure S10); therefore, equilibration of initial configurations at service temperature (around 300 K) is not feasible. Besides, there is a high chance of being kinetically trapped in unfavorable configurations if one performs equilibrations well above T_g followed by a (direct) rapid cooling to 300 K, similar to many polymer glasses[1]. Therefore, we used a "sub-T_g relaxation" protocol, as was previously used for similar polymers in refs [2,3]. Thus, we use an intermediate annealing at 500 K (just below T_g) between the equilibration well above T_g (at 900 K) and at 300 K. A schematic of this procedure is depicted in Figure 1b (in the main manuscript).

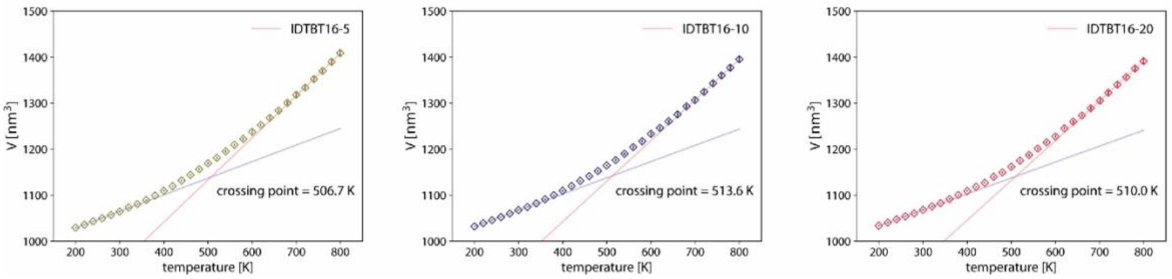

Figure S10. Volume-temperature curves for the three polymer models. The intersection of fitted lines on the data well-above (700-800 K) and well-below (200-300 K) of the transition is used to estimate T_g. The cooling rate for all simulations is 5 K/ns. The standard deviation of volume at each temperature step is shown with vertical error bars.

π-π interaction analysis

Similar to our previously implemented method[2], we defined the plane spanned by the heavy atoms of each IDT, and BT fragments (through a least squares fitting) and recognized π-π interaction between them that satisfy the following criteria: 1) the angle between two planes' normal vectors is smaller than 10° (parallel fragments), 2) π-π distance (marked as $D_{\pi\text{-}\pi}$ in Figure S11a) is smaller than 0.5 Å, and 3) the horizontal distance between the centre of geometry of each parallel pair (marked as H_{COG} in Figure S11a) is smaller than 5.0 Å. Figure S11b shows the distribution of $D_{\pi\text{-}\pi}$ as calculated for 150 π-π coupled BT fragments obtained from 5 independent simulations snapshots (each extracted from a different annealing cycle). As shown, the distribution peaks around $D_{\pi\text{-}\pi} \approx 3.6$ Å, a typical distance for π-π interaction.

It should be note that possible π-π interactions between the central six-carbon ring of IDT (not the whole fragment) was also explored to check possible crossings between IDT fragments and the number of π-π interactions between them were as rare as the IDT-IDT π-π interactions (<0.005 of total IDT fragments in the simulation box).

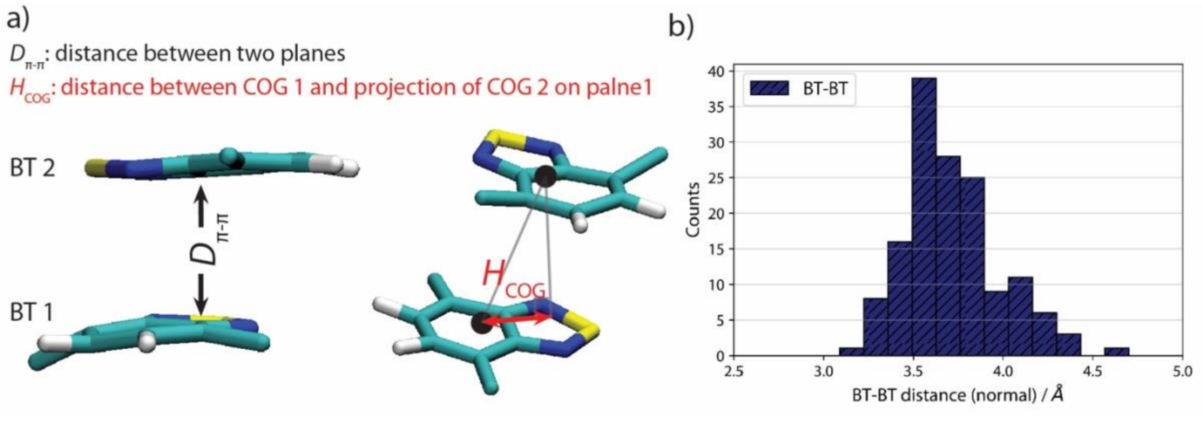

Figure S11. (a) Illustration of $D_{\pi-\pi}$ and H_{COG} for one example of BT-BT in π-π interactions. (b) distribution of $D_{\pi-\pi}$ as calculated for 150 π-π coupled BT fragments in 5 independent simulations snapshots (each extracted from a different annealing cycle).

The code which identifies and calculates π-π interactions and examples of required coordinate and index files for this calculation are provided in https://github.com/HMakkiMD/IDTBT.

Sidechain effect

To confirm the origin of BT-BT crossings, we generated IDTBT chain models with different sidechain lengths, i.e., IDTBT8-5, IDTBT4-5, and IDTBT1-5 (see monomer structures in Figure S12 a), and equilibrated these polymer models in a similar way as IDTBT16-5. Then, the number of BT-BT π-π interactions and θ_{ij} for all models were calculated. As shown in S12 b and c, the number of π-π interactions increases and the distribution of θ_{ij} tends towards 0 ° as the sidechain length is shortened. This reconfirms that the reason behind relative perpendicular orientation of BT-BT crossings is the steric hinderance of the bulky sidechains.

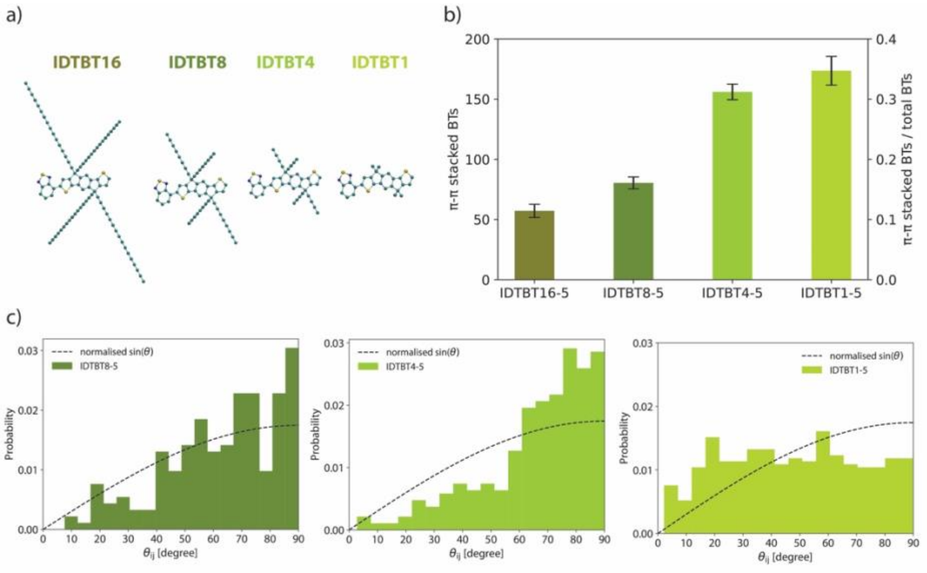

Figure S12. (a) IDTBT monomer models with varying sidechain lengths. (b) Number of BT-BT π-π interactions for different polymer models with varying sidechain lengths. (c) Distribution of θ_{ij} for different polymer models with varying sidechain lengths.

We calculated the torsional angle distribution and the chain orientation (against z axis) for equilibrated models of IDTBT16-5 and IDTBT1-5, see Figures S13 and S14, respectively. As shown, the change in the relative orientation of chains (as a result of shortening the sidechains) does not influence the distribution of torsional angles, however, the packing of chains lead to an obvious deviation from random orientation- a common observation for semi-crystalline polymers. It is worth noting that according to the reports in the literature[4], it is not possible to synthesize IDTBT polymer with sidechains shorter than 4 carbon length (IDTBT4) due to the solubility problem of the monomers during polymerisation, and our analysis on the hypothetical polymers (e.g., IDTBT1) has been only done to unravel the explicit role of sidechains on IDTBT morphology.

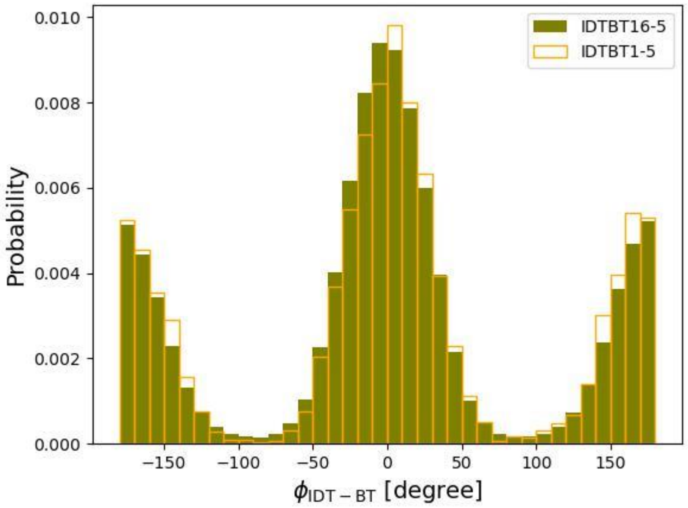

Figure S13. IDT-BT torsional angle distribution for IDTBT16-5 and IDTBT1-5 at 300 K.

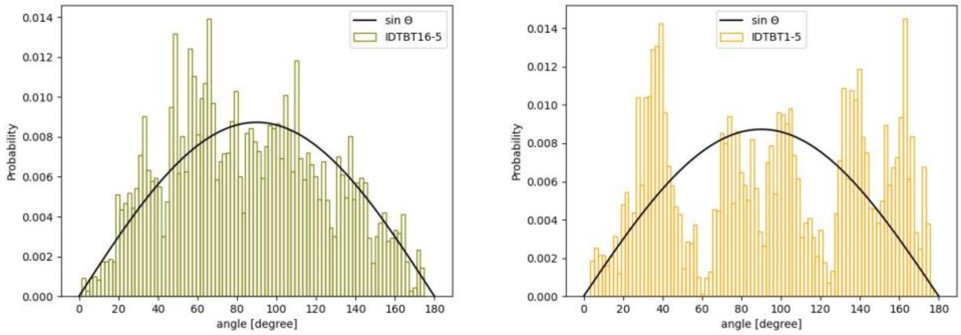

Figure S14. The distribution of angles between chain directors (a vector connecting the two end of chains) with the z axis for IDTBT16-5 (left) and IDTBT1-5 (right) models. The normalised sin θ function is shown evaluate the randomness of distributions.

S3. X-ray Scattering Pattern Calculation

We use Debye scattering equation (SE5)[5] to calculate the X-ray scattered intensity of polymer models.

$$I_q = \sum_i \sum_j f_i f_j \frac{\sin(qr_{ij})}{qr_{ij}} \qquad \text{SE5}$$

where I_q is the scattering intensity, f is atomic form factor, q is the magnitude of the scattering vector, and r_{ij} is the distance between atoms i and j. We employed a distance-histogram approximation [4] to avoid calculation of expensive sine function for each atomic pair. Therefore, we split computations into two steps: (i) a histogram of distances for each pair of atoms (excluding hydrogen atoms) is calculated and (ii) the Debye formula treats distances in each histogram bin at once so that the Debye formula (assuming a mono atomic system) can be written as SE6 (derivation can be found in https://debyer.readthedocs.io/en/latest/).

$$I_q = f^2 \left(N + 2 \sum_k n_k \frac{\sin(qr_k)}{qr_k}\right) \qquad \text{SE6}$$

where N is the number of atoms (excluding hydrogen) in the simulation box, n_k and r_k are the are the number of pairs and the distance corresponding to the k-th bin.

The code by which the scattering patterns were calculated is provided here https://github.com/HMakkiMD/IDTBT.

S4. Quantum Chemical Calculations

Sampling Procedure for Electronic Structure Calculations

Chain conformations for electronic structure calculation were extracted periodically throughout molecular dynamics simulation as snapshots, which were deemed to be sufficiently uncorrelated from one another at a simulation time difference of one annealing cycle (25 ns total in the NPT ensemble with 10 ns at 900 K, 10 ns at 500 K, 2 ns at 300 K, and 3 ns for temperature ramping). 5 snapshots for each system were considered, with equivalent sampling for chains of varying lengths determined according to the following equation,

$$T_S = (N_c \cdot L \cdot N_S) \tag{SE7}$$

where T_S is the total sampling, N_C is the number of chains in the simulation box, L is the chain length (in number of monomers), and N_S is the number of snapshots. If we set T_S to 2500, and N_S is 5, then the values of N_C for IDTBT16-5, IDTBT16-10, and IDTBT16-20 are 100, 50, and 25, respectively.

Some approximations were necessary for efficient computation of the electronic structure. Firstly, the $-C_{16}H_{33}$ side-chains modelled in MD simulation were truncated to $-CH_3$ groups in order to significantly reduce computational expense with negligible effect on the electronic structure due to the dominant contribution from the backbone. A second approximation was the treatment of the electronic structure of individual chains separately, as the overall influence of coupling between chains is expected to be minimal. To consider the electrostatic environment of each chain, all monomers containing a charged atom within 20 Å of a charged atom on the central chain were included as point charges in the calculation, where the charges on each atom were the same as those used for the force field and therefore balanced. The B3LYP/3-21G level of theory was used as implemented in Gaussian16 software for these calculations.

Density of States

The density of states (*DOS*) was computed for all one-electron states according to the abovementioned sampling procedure. We define the DOS here similarly as the per chain per monomer *DOS*,

$$\rho_i(E) = \sum_m g\left(E - E_i^{(m)}\right)/M \tag{SE8}$$

where $E_i^{(m)}$ is the energy of molecular orbital m for chain i, M is the number of monomers in the chain, and g denotes a Gaussian approximation for the Dirac delta function. The bulk *DOS*, $\rho_b(E)$ (shown as *DOS*(E) in the manuscript) is then an average over the per chain per monomer *DOS* for all chains, where N_C is the total number of chains considered. A Gaussian broadening parameter of 0.025 eV was used for computation of the *DOS*.

$$\rho_b(E) = \sum_{i=1}^{N_c} \rho_i(E)/N_c \tag{SE9}$$

Figure 5c of the main paper shows a bulk partial density of states, *PDOS*(E), for IDTBT, which is a projection of the contributions of IDT and BT fragments to the total *DOS*. The per chain per monomer *PDOS* for a particular fragment is calculated as follows,

$$\rho_{f,i}(E)=\sum_{m} P_f^{(m)}\cdot g\left(E-E_i^{(m)}\right)/M \qquad \text{(SE10)}$$

where $P_f^{(m)}$ is the weight of a molecular orbital *m* on a fragment *f* and is calculated by equation SE11, with partitioning of the chain into fragments.

$$P_f^{(m)}=\sum_{\substack{\text{i on f}\\ \text{j on all}}} C_i^{(m)} S_{ij} C_j^{(m)} \qquad \text{(SE11)}$$

where $C_i^{(m)}$ is the coefficient of atomic orbital i on molecular orbital *m* and S_{ij} is the overlap between atomic orbitals *i* and *j*.

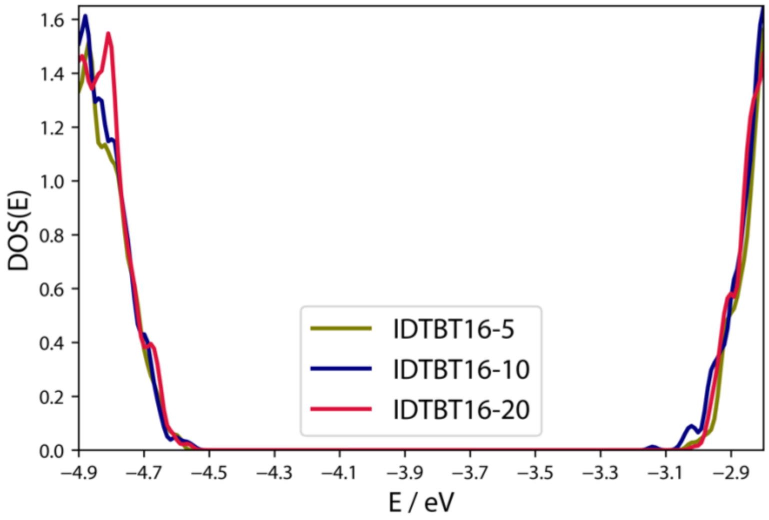

Figure S15. Electronic bandgap of the three polymer models calculated by our QC/MD method.

Transfer Integral Calculations

Transfer integrals were computed as a measure of the coupling between 150 BT crossing points, which we deem to be an interchain BT pair in a stacking configuration. We define a stacked pair as one where both the vertical and horizontal distance between the centres of geometry of each fragment is smaller than 5 Å and the angle between normal vectors of planes fitted to the heavy atoms of each is smaller than 10°. BT fragments were isolated by cutting at the carbons connected at either side (inclusive), and made whole by replacing these atoms with hydrogen.

Transfer integral calculations were performed using in-house Python code according to the method presented in ref[6], using the definition,

$$J_{ij}=\left\langle \varphi_i \middle| \hat{F} \middle| \varphi_j \right\rangle \qquad \text{(SE12)}$$

where φ_i and φ_j are the unperturbed HOMO orbitals of the monomers and $\hat{F}$ is the Fock operator of the molecular dimer. Electronic structure calculations were carried out at the B3LYP/3-21G* level of theory as implemented in Gaussian16.

References

(1) Barrat, J. L.; Baschnagel, J.; Lyulin, A. Molecular Dynamics Simulations of Glassy Polymers. *Soft Matter* **2010**, *6* (15), 3430–3446. https://doi.org/10.1039/b927044b.

(2) Makki, H.; Troisi, A. Morphology of Conducting Polymer Blends at the Interface of Conducting and Insulating Phases: Insight from PEDOT:PSS Atomistic Simulations. *J. Mater. Chem. C* **2022**, *10* (42), 16126–16137. https://doi.org/10.1039/d2tc03158b.

(3) Keene, S. T.; Michaels, W.; Melianas, A.; Quill, T. J.; Fuller, E. J.; Giovannitti, A.; McCulloch, I.; Talin, A. A.; Tassone, C. J.; Qin, J.; Troisi, A.; Salleo, A. Efficient Electronic Tunneling Governs Transport in Conducting Polymer-Insulator Blends. *J. Am. Chem. Soc.* **2022**, *144* (23), 10368–10376. https://doi.org/10.1021/jacs.2c02139.

(4) Wadsworth, A.; Chen, H.; Thorley, K. J.; Cendra, C.; Nikolka, M.; Bristow, H.; Moser, M.; Salleo, A.; Anthopoulos, T. D.; Sirringhaus, H.; McCulloch, I. Modification of Indacenodithiophene-Based Polymers and Its Impact on Charge Carrier Mobility in Organic Thin-Film Transistors. *J. Am. Chem. Soc.* **2020**, *142* (2), 652–664. https://doi.org/10.1021/jacs.9b09374.

(5) Debye, P. Zerstreuung von Röntgenstrahlen. *Ann. Phys.* **1915**, *351* (6), 809–823.

(6) Domcke, W. Theory of Resonance and Threshold Effects in Electron-Molecule Collisions: The Projection-Operator Approach. *Phys. Rep.* **1991**, *208* (2), 97–188. https://doi.org/10.1016/0370-1573(91)90125-6.
